# Refractory secondary hyperparathyroidism in waiting list for parathyroidectomy: who we should operate first in a quaternary hospital in Brazil regarding survival

**DOI:** 10.1080/0886022X.2019.1590210

**Published:** 2019-04-03

**Authors:** Lia Roque Assumpção, Isadora de Paula Ramos, Gerson Nunes da Cunha, Cid Manso de Mello Vianna, Maria Cristina Araújo Maya, Denizar Vianna Araújo

**Affiliations:** a Department of Surgery, Rio de Janeiro State University, Rio de Janeiro, Brazil;; b Rio de Janeiro State University, Medical Sciences Post-Graduation Program, Rio de Janeiro, Brazil;; c Planning and Health Policy Department, Rio de Janeiro State University, Rio de Janeiro, Brazil;; d Department of Internal Medicine, Rio de Janeiro State University, Rio de Janeiro, Brazil

**Keywords:** Hyperparathyroidism, secondary, parathyroidectomy, survival analyses, waiting list

## Abstract

**Background:** Few centers in Brazil perform parathyroidectomy (PTX) for recalcitrant secondary hyperparathyroidism (SHPT) generating a long queue. There is little data regarding prioritize criteria besides chronological order and survival.

**Objectives:** To determine the difference of clinical and laboratory factors between PTX patients and those who remained in the line despite the need for surgery and their survival.

**Methods:** A retrospective cohort study was conducted in a quaternary hospital in Brazil, where 43 patients with PTX indication due to severe SHPT were followed from 2009 to 2016. While 31 patients underwent PTX, 12 remained in the queue. Data on clinical and laboratory factors were collected for comparison and Kaplan–Meier and Cox regression survival analysis were used.

**Results:** PTX group was younger (40.9 vs. 49.3 years, *p* = .03), had higher PTH levels (2578 vs. 1937 pg/ml, *p* = .01) and higher CaxP product (62 vs. 47.5, *p* = .02). There were no percentage differences between groups of fractures, calciphylaxis and other complications due to SHPT. Patients who were not operated had a worst overall survival (5 y 62.2% vs. 96.7%, *p* = .04) with a HR for death of 8.08 (*p* = .07, PTX as a TVC). Other variables associated with decreased survival included a history of previous myocardial infarction (HR: 10.4, *p* = .01) and age per additional year (HR: 1.09, *p* = .02).

**Conclusions:** Patients with severe SHPT are at increased risk of death while waiting for PTX. Clinical events like fracture were not used to prioritize patients beyond consecutive order. Therefore, optimizing priority criteria for PTX may result in improved survival in this population.

## Background

Based on a chronic kidney disease (CKD) 2016 census, there are 113 000 patients currently on hemodialysis (HD) in Brazil. Among these patients, 18% were found to have parathormone (PTH) levels above 600 ng⁄ml, characterizing secondary hyperparathyroidism (SHPT) development [1]. Compared with patients on HD but with no SHPT, patients with SHPT have a 21% increase in mortality [2].

According to the Kidney Disease Outcomes Quality Initiative (KDOQI), parathyroidectomy (PTX) is indicated when PTH levels are beyond 800 ng⁄ml, which is considered severe SHPT, although there is lack of high-level evidence to support this practice [3]. The international guidelines from Kidney Disease: Improving Global Outcome (KDIGO) also support PTX in CKD patients grade 3–5 with severe SHPT [4,5].

In Brazil, 83% of CKD patients rely exclusively on the Brazilian public health system. As a result, access to care is limited with only 24% of stage 5 CKD patients listed for kidney transplant programs and with inadequate access to effective drugs for the treatment of SHPT such as paricalcitol and cinacalcet [1]. In addition, only a few centers within the public health system network are suitable to perform PTX in patients with severe SHPT. In 2014, a Delphi Panel was conducted with Brazilian SHPT specialists, which estimated that 70% more patients are designated to PTX, compared to those who were preferably suited to the procedure [6]. Therefore there is a waiting list due to an unbalance between PTX demand and surgical resolution. This has worsened since a National Cancer Access Law was approved in 2012, granting treatment within 60 days of diagnosis for oncological patients, making their PTX even more difficult to be achieved in the public health system scenario [7].

Surgical waiting lists typically follow chronological order according to date of entry in a First in First out model (FIFO) [8]. In patients with refractory SHPT, surgery can potentially be prioritized due to the intensification of symptoms severity, such as incapacitating pain, or major events such as fractures or calciphylaxis. In a national level, there are data showing increased mortality for those with severe SHPT on the waiting list when compared to those who were operated [9]. However, prioritization criteria out of FIFO order to decrease overall mortality have yet to be established for this population. The present study aimed to determine the difference of clinical and laboratory factors between the operated group and those who remained in the line despite the need for surgery. In addition, it aimed to determine the survival benefit of PTX and factors associated with survival in patients with severe SHPT while in the waiting list.

## Methods

### Study design

A retrospective cohort observational study was conducted in a single quaternary university hospital in Rio de Janeiro (Pedro Ernesto University Hospital, Rio de Janeiro State University), Brazil. Patients with refractory SHPT were sent to a surgical appointment and were listed according to FIFO order. Clinical and laboratory criteria were evaluated periodically and if there were deterioration of their clinical status (like fractures, calciphylaxis or excruciating pain that prevented them from walking or doing everyday self-care), the case was discussed during the weekly grand rounds and it was decided whether or not it was worth it to become priority case outside of the FIFO order.

Inclusion criteria of the participants were: age >18 years old; having SHPT and be in HD treatment >3 years; utilization of phosphorus binders with or not calcitriol and or not cinacalcet and not responding to their use; PTH >800 pg/ml, follow up >12 months. Exclusion criteria were: having PTX or kidney transplant previously, tertiary hyperparathyroidism and persistence of SHP due to an ectopic or supernumerary parathyroid gland after first PTX. From 2009 to 2016, 43 patients fulfilled the aforementioned criteria and they were divided in two groups: patients who had PTX performed (31) and those who remained on the waiting list (12, queue) even though surgery was desirable.

### PTX

We performed total PTX with auto-implant of fragments of the best macroscopically retrieved gland, with less nodular degeneration, after frozen pathology confirmation. Auto-implant was done immediately on the same forearm (brachioradialis muscle) of their HD arteriovenous fistula for all patients. Implant pouches are marked with non-absorbable sutures.

### Data collection

Demographics, clinical and laboratory variables were collected before PTX and last consultation available for those who were in the queue group. They were age, gender, serum calcium and phosphorus, PTH, alkaline phosphatase, dialysis vintage, events as fractures, calciphylaxis (presence of any painful deep skin ulceration not healing and not trauma related), ectopic calcifications (presence of echocardiogram showing any cardiac/valve calcification; CT scan, MRI or X-ray showing any muscle or arterial calcification), myocardial infarction (MI) (presence of typical ischemic-type chest discomfort, elevated cardiac troponin testing and ST-segment elevation, depression, T wave inversion or pathological Q-waves on the ECG), waiting time on the list, comorbidities as diabetes, hypertension and symptoms like excruciating pain, bone deformity and depression. Follow up time was considered as the interval between first surgical consultation until the occurrence of the interested event (death) or until the subject was censored (lost to follow up after a minimum period of 12 months monitoring and maximum until December of 2016 without death or getting kidney transplant surgery after PTX).

### Statistical analyses

After gathering variables in chart reviews and active patient follow up search, a database was assembled, and statistical analysis was performed using SPSS 18.0 (SPSS Inc., Chicago, IL) and STATA 12.0 (State College). Considering the normality of the continuous variable either *t*-test or the Wilcoxon signed ranks test was performed. Categorical variables expressed as percentages were compared using the chi-square test. In all cases, a two-sided *p* values <.05 were considered to be statistically significant. Survival analyses were achieved using Kaplan–Meier methods with a log-rank test to see differences in overall survival. A Cox proportional hazard regression model was done to calculate hazard ratios of death and 95% confidence intervals. For a second survival analyses, PTX was included as a time-varying covariate in other to exclude ‘‘immortal time’’ bias, using the PTX date as the beginning time for PTX group.

This study was approved by the local ethics committee for research and received national research ethics registry approval number CAAE 40831114.0.0000.5259.

## Results

### PTX vs. queue

The 31 operated patients within the assessed period were younger (mean difference of 8.3 years, *p* = .03), had higher PTH (mean 2578 vs. 1937 pg/ml, *p* = .01) and calcium-phosphorus product (mean 62 vs. 47.5, *p* = .02) when compared to the 12 patients from queue group. However, PTX patients had a lower waiting time for surgery (median 10 months for PTX vs. 26 months for queue, *p* < .01). Patients who remained on the line tend to use more cinacalcet than those who had surgery (58.3% vs. 12.9%, chi-square *p* = .03). There was no more statistical difference between the PTX and queue groups regarding other clinical and laboratory factors as dialyzes vintage, fractures or alkaline phosphatase levels as shown in Table 1.

**Table 1.** Demographics.

Clinicopathologic patient characteristics	Patients		
Number of patients	PTX *n* = 31	Queue *n=* 12	*p* value
Male	64.5%	58%	.71
Age*	40.9 (12.1)	49.3 (14.6)	.03
Queue time in months**	10 (5–21)	26 (14–56)	.004
Dialyses vintage in years**	10 (8–13)	9 (6–10)	.22
Cinacalcet^#^	12.9%	58.3%	.03
Diabetes	0%	8.3%	.1
Hypertension	90.3%	83.3%	.52
Fracture	41.9%	41.7%	.99
≥2 Fractures	9.7%	0%	.16
Ectopic calcification	41.9%	33.3%	.49
Calciphylaxis	3.23%	16.7%	.14
MI	9.7%	25%	.23
Bone pain	96.8%	100%	.53
Bone deformities	70.9%	75%	.91
Brown tumor	22.6%	25%	.91
Muscle atonia	70.9%	75%	.64
Pruritrus	19.4%	42%	.21
Depression	25.8%	50%	.33
PTH* (pg⁄mL)	2578 (776)	1937 (661)	.01
AFP* (U/L)	1325 (809)	993 (505)	.15
CaxP (product)*	62 (15)	47.5 (6.3)	.02

*Mean with standard deviation, **median with interquartile range, ^#^used only after 2013.

### PTX morbidity

**Figure 1.**
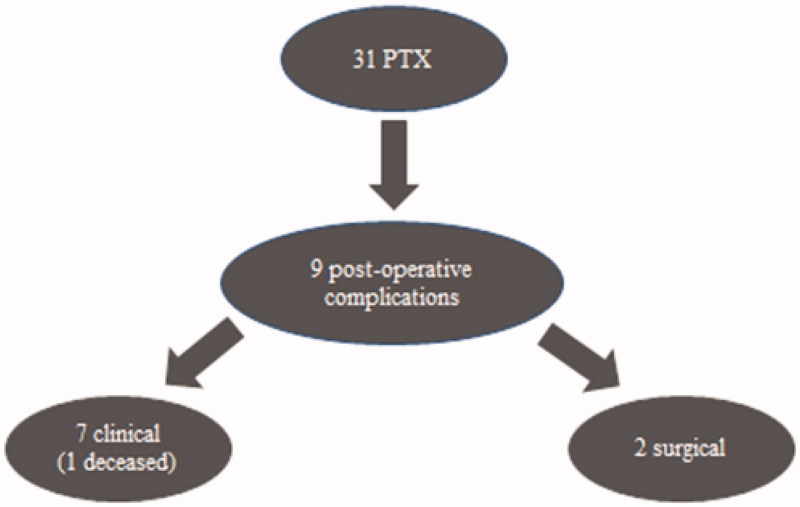
Surgical morbidity.

Of the operated patients, 21(67.7%) of them presented with pre-operative surgical risk categorized as ASA III, even though 27 (87.1%) needed an ICU for immediate post-operative care [10]. After PTX there was a significant drop of PTH levels from mean 2578 to 121 ng⁄ml (paired *t*-test, *p* < .001). Length of stay after surgery was a median of 11 days (IQ range: 8–15 days), where 100% of them had post-operative hungry bone syndrome. Post-operative morbidity was 29%, exposed in Figure 1. Seven patients had clinical cardiac (57%) and pulmonary (43%) complications and one of those patients deceased on the twenty-fourth day of the hospital stay. Two patients had surgical complications: one wound infection and one oral bleeding from a mandibular brown tumor that required surgical hemostasis.

### Survival

**Figure 2.**
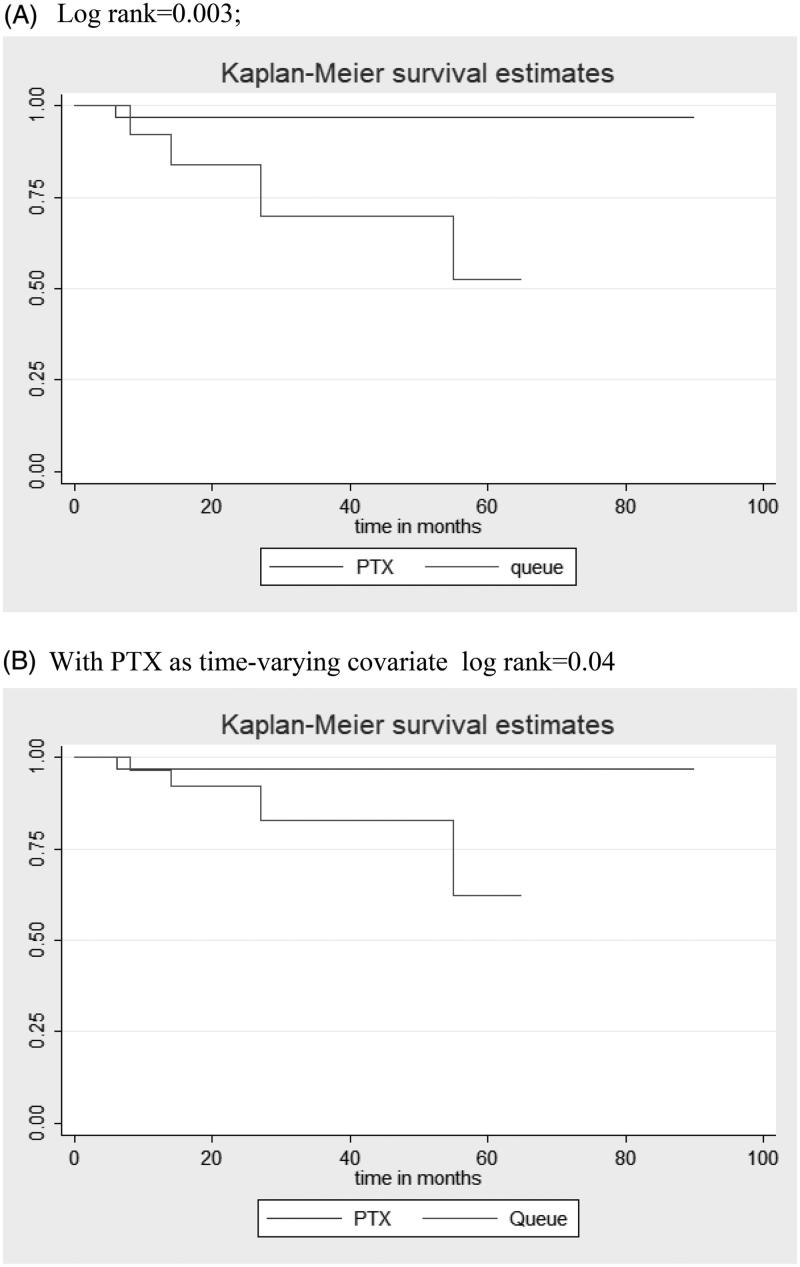
Kaplan–Meier curves. (A) Log-rank = 0.003; (B) With PTX as time-varying covariate log rank = 0.04.

Patients who remained waiting for surgery in the queue group had lower survival in relation to the PTX group (5 years survival 62.2% vs. 96.7%, log-rank test’s *p* = .04 with PTX as a time-varying covariate), curves shown in Figure 2. Median follow up time of the entire cohort was 56 months (IQ range 27–75 months).

In univariate Cox regression for survival analysis, patients on the line have more 13.5 times chance of dying (*p* = .02), as well as patients who had previous MI (HR: 10.4, *p* = .01) and also older patients (HR: 1.09 for each additional year, *p* = .02). Patients who used cinacalcet (after 2013) had a risk reduction of death when compared to those who did not use (HR: 0.16, *p* = .05), this protective effect persisted when controlled for MI. Expressed in Table 2, values of hazard ratios with and without considering ‘immortal time’ bias can be compared. When considering PTX as a time-varying covariate, there was a trend of increase hazard death risk for queue group, but the coefficient value was lower and there was a lost a statistical significance (HR: 8.08, *p* = .07), log-rank increased in value from 0.003 to 0.04 but maintained worst survival (Figure 2(A,B)). None of the variables were independent risk factors when bivariate Cox regression was performed using MI and age, presented in Table 2.

**Table 2.** Cox regression for survival analyses.

Variables	Hazard ratio	95%CI	*p* value
Univariate			
Queue	13.51	1.48–122.93	.02
Queue (PTX as TVC)*	8.08	0.83–78.18	.07
Age (continuous)	1.09	1.01–1.18	.02
PTH	0.99	0.99–1.00	.49
CaxP (product)	0.94	0.83–1.08	.38
Hypertension	1.59	0.18–14.34	.68
Fractures	2.06	0.34–12.37	.43
Calciphylaxis	2.79	0.31–25.08	.34
Ectopic calcification	1.07	0.18–6.45	.94
MI	10.36	1.73–62.21	.01
Bone deformities	1.31	0.15–11.81	.81
Brown tumor	2.58	0.42–15.64	.3
Dialyses vintage (continuous)	0.98	0.74–1.28	.86
HD >10 years	1.78	0.29–10.67	.53
Cinacalcet	0.16	0.03–0.98	.05
Bivariate			
Age (continuous)	1.05	0.97–1.15	.22
MI	4.87	0.58–40.55	.14

*TVC: time-varying covariate.

## Discussion

### PTX vs. queue

Albeit kidney transplant (KTx) could be a successful way out of the line for PTX, not all patients on the list are able to get it. Interestingly, Brazil lies within the countries worldwide that mostly perform KTx in absolute numbers. Conversely, access to this procedure does not exceed 25 per million of the population (pmp) whereas other countries like USA, Portugal and Croatia have rates of KTx that achieve >50 pmp [11,12]. Countries or regions who had similar difficult numbers of access to KTx, economical problems with medicine purchase and surgical treatment for SHPT might profit from the question raised in this study, who we should be operating first.

The waiting list for PTX in Brazil constitutes an organizational challenge within the public health system of CKD patients with severe SHPT. Particularly in Brazil a dialyzes outpatient census revealed that 10% of patients were categorized as having highly severe SHPT (PTH >1000 ng⁄ml) and 57% of PTX was performed within the first year of queue admittance. Despite this fact, 23% of patients did not have referential PTX centers available for reference. In Brazil, only 68 hospitals perform the procedure and 41% of them belonged to University Hospitals [13]. Our results corroborate with the aforementioned census, since our patients received PTX with a mean time of 10 months after being admitted to the line. Nevertheless, serious clinical events as fractures, calciphylaxis, bone deformities and excruciating pain, that could possibly alter the FIFO order of the line, were not found in greater percentages on the PTX group. Operated patients were younger, had higher PTH and calcium-phosphorus product, biochemical markers of disease burden, and the majority needed to be held in ICU for immediate post-operative care. Hence, maybe logistic factors like ICU beds and long-term hospital stay availability might influence as a restraint management of this scenario of patients [14]. Restriction to post-operative ICU bed access limited not only our surgical capacity for performing PTX, but also probably created on the queue group a longer waiting time when compared to those who succeed in getting their surgery. Therefore, rather than exclusively considering FIFO order, we are probably using more opportunity to operate (i.e., patient number two on the FIFO list does not need an ICU bed for post-operative care tends to get surgery first if there is a room to operate him but no ICU bed availability, when compared to patient number one for FIFO but who needs an ICU bed) and also taking into account deterioration of their clinical status respecting their order first placed on the FIFO way. Another factor to be considered was a national Brazilian Cancer Law, approved in 2012, that granted access for cancer treatment 60 days after diagnoses in the public health system. That decreased the amount of inpatient hospital vacancy for patients with severe benign diseases such as SHPT, since there was no increase in beds within complex hospitals [7,14].

Calcimimetics agents were incorporated nationally in 2015, but their use was authorized after a successful lawsuit by a group of patients since 2013, and its use by our cohort was casually interrupted by lack of governmental distribution [6]. Though not constantly available for all, cinacalcet use could have resulted in better control of severe SHPT in the queue group, with some overall survival impact of our whole cohort (87% protective effect for death in univariate analyses). Dutch data stated that cinacalcet use could procrastinate surgery for up to two years after CKD evolve to SHPT, but as its use was not homogenous in our cohort, this effect was impossible to be quantified in the present study [15].

### PTX morbidity and mortality

After PTX, there was a drop of more than 95% of pre-operative PTH values within 48 h, displaying good quality of the procedure (total PTX with auto-implant), as recommended by Montenegro et al. [16]. Even though recent data proposed that tailored resection of subtotal PTX with drop of 75–80% PTH achieved good outcomes, with shorter length of stay after PTX when compared to ours (median of 5 vs. 11 days) [17]. Our length of stay characterized that 100% of our patients presented with hungry bone disease requiring longer time needed for intravenous calcium replacement despite oral calcitriol and calcium intake.

Specifically considering severe SHPT, observational studies already reported increased mortality in this population, induced by toxic PTH concentrations, which can lead to immunological, hematological, metabolic and cardiovascular alteration [1,18]. In the USA, 30 days mortality rate is about 3.1%, likewise in this study, we report a mortality rate of 3.2% within the operated patients [19,20]. Our patients had severe comorbidities as consequences of a mean 10 years history of dialyzes when surgery was performed, this fact can contribute to higher surgical morbidity. Almost one-third of our PTX patients had some level of complication during the following days after surgery, the majority of them due to pulmonary and cardiac associated problems. One patient had to be operated because of bleeding outside the surgical site (mandibular brown tumor). The association of the surgical trauma and thoracic deformation from the extensive bone disease and pronounced cardiovascular disease burden previous to the PTX could explain the occurrence of such complications. Similar rates of surgical complication related to early death were predominantly by cardiovascular infectious problems [19–21]. Despite morbidity, there is recent data from Brazilian and Taiwan reporting quality of life improvements in seven out of 8 SF36 criteria one year after PTX, showing the benefits of surgery concerning patient perceptions [22,23].

### PTX and long-term survival

About improving survival, latest meta-analysis demonstrated a decrease after PTX in all-cause mortality by 28%–36%, and by 37%–41% to cardiovascular-specific mortality causes [24,25]. This data validates our data for overall cumulative survival benefit regarding PTX patients. Time urges to severe SHPT patients waiting on the line, as Brazilian’s mortality to be in line was previously estimated to be 4.13 times higher according to a single center report in Sao Paulo [8]. We also found an increase of death risk in univariate Cox regression, where patients who remained in line were 13.5 times more likely to die, even though we had a large confidence interval to this point estimate due to few patients enrolled in the study. When PTX was included in survival analyses as a time-varying covariate, ‘immortal bias’ was excluded improving survival for the queue group considering comorbidities impact until PTX was performed. In this second analyses, HR to be waiting in line dropped to a point estimate of 8.1 with one crossing over the confidence interval. It was higher than the one from Sao Paulo, still, it could be explained by greater severity of clinical and laboratory characteristics among our cohort. Admirably, known risk factors that would stratify patients to get surgery first, as fractures, calciphylaxis and bone deformities that impact their functional capacity, were not mortality predictors. None of the factors were independent predictors in a bivariate Cox regression, this happened probably due to collinearity of the factors. Again cardiovascular disease burden played an effect in this study, when controlled for PTX, previous MI increased in 12 fold chance of death. Goldstein et al. described that younger patients tended to be more operated, we also found this result [8]. In univariate Cox regression there was an increased chance for mortality of 9% per year (Goldstein was 4%), each year a patient gets older. This data can lead to more sophisticated larger cohorts analysis, considering that proportional risk of comparable groups could not be constant through time and breaking Cox proportional hazard assumption could result in closer to real life inference of risk [26].

## Limitations

The present study has limitations such as the small number of patients in whom PTX was performed and those on queue group, both with more than one year of follow up, and not quantifying cause-specific death rates, therefore not stratifying cardiovascular mortality rates. As patients arrived for queue admittance with already very severe SHPT, we don’t know what baseline cardiovascular disease burden is increased by each year of more HD in an uncontrolled disease. This fact could have some influence on patient selection to PTX towards healthier patients. We did not adjust for this confounding factor, already identified in a previous study [27].

This research intended to estimate waiting time effect for PTX in severe SHPT mortality, considering a public health system where FIFO rules together with disease worsening criteria to manage the queue, respecting ethical and fair access. Unfortunately, we were not able to estimate (in significant independent hazard ratios) objectives clinical and laboratory risk factors capable of being used to prioritize PTX besides FIFO, in a follow-up time larger than one year. Events like fracture and calciphylaxis, for example, did not have statistical inference for mortality or surgery. However the previous medical history of MI and age were predictors for death in this cohort and therefore attention to this group in granting better access for surgery should be addressed.

Our cohort was represented by an extreme group of patients with severe SHPT which waiting time for PTX tend to belong within our public health system, after a long period of dialyzes vintage. Difficulties for referral and surgical resolution in good time frame can affect patient survival and also put them in worse clinical condition when surgery is performed, due to natural history of the disease. Factors that will prioritize patients out of FIFO order should be further evaluated and selection for elective surgery still needs constant updates on patient disease burden. Optimizing surgical queue for PTX and sanctioning better access to treatments like cinacalcet and KTx should minimize the high mortality in long term for this group of patients.

## Conclusion

Patients with severe intractable SHPT are at an increased chance of death while waiting for PTX. Expected risk factors such as fractures, calciphylaxis and calcium-phosphorus product were not predictors for mortality in our scenario and could not be used broadly to pass on one patient in front of the other. A case by case surgeon analyses still needs to be performed in order to manage the PTX line. In contrast, previous MI and each year increased of age were associated with poor survival in this cohort, yet none of the factors were independent predictors. For that reason, decreasing PTX queue time with clear upgrade criteria may improve patient survival in this population.

## References

[1] Sesso RC, Lopes AA, Thomé FS, et al. Brazilian chronic dialysis survey 2016. J Bras Nefrol. 2017;39:261–266. 29044335 10.5935/0101-2800.20170049

[2] Tentori F, Blayney MJ, Albert JM, et al. Mortality risk for dialysis patients with different levels of serum calcium, phosphorus, and PTH: the Dialysis Outcomes and Practice Patterns Study (DOPPS). Am J Kidney Dis. 2008;52:519–530. 18514987 10.1053/j.ajkd.2008.03.020

[3] National Kidney Foundation. K/DOQI clinical practice guidelines for bone metabolism and disease in chronic kidney disease. Am J Kidney Dis. 2003;42:S1–S201. 14520607

[4] Kidney Disease: Improving Global Outcomes (KDIGO) CKD-MBD Work Group. KDIGO clinical practice guideline for the diagnosis, evaluation, prevention, and treatment of Chronic Kidney Disease-Mineral and Bone Disorder (CKD-MBD). Kidney Int Suppl. 2009;113:S1–S130. 10.1038/ki.2009.188 19644521

[5] Isakova T, Nickolas TL, Denburg M, et al. KDOQI US commentary on the 2017 KDIGO clinical practice guideline update for the diagnosis, evaluation, prevention, and treatment of chronic kidney disease-mineral and bone disorder (CKD-MBD). Am J Kidney Dis. 2017;70:737–751. 28941764 10.1053/j.ajkd.2017.07.019

[6] Araújo DV, Amaral LM, Guersoni AC, et al. Secondary hyperparathyroidism treatment costs with cinacalcet or PTX, for uncontrolled patients with conventional clinical therapy under Brazilian Public Health System perspective. J Bras Econ Saúde. 2017;9:54–61.

[7] LEI 12.732/2012 (LEI ORDINÁRIA) 11/22/2012, Diário Oficial da União - Seção 1 - 23/11/2012, Page 1. [cited 2018 Sep 4] Available from URL: http://www.planalto.gov.br/ccivil_03/_ato2011-2014/2012/lei/l12732.htm

[8] Tan KW, Wang C, Lau HC Improving patient flow in emergency department through dynamic priority queue. 2012 IEEE International Conference on Automation Science and Engineering (CASE), Seoul; 2012 p. 125–130.

[9] Goldenstein PT, Elias RM, Pires LFC, et al. Parathyroidectomy improves survival in patients with severe hyperparathyroidism: a comparative study. PLoS One. 2013;8:e68870. 23940515 10.1371/journal.pone.0068870 PMC3734286

[10] Saklad M Grading of patients for surgical procedures. Anesthesiology. 1941;2:281–284.

[11] Garcia GG, Harden P, Chapman J, World Kidney Day Steering Committee The global role of kidney transplantation. Nephrol Dial Transplant. 2013;28:e1–e5. 2013. 22822091 10.1093/ndt/gfs013

[12] Global Observatory on Donation and Transplantation. Organ donation and transplantation activities. 2015 [cited 2018 Sep 4] Available from URL: http://www.transplant-observatory.org/2015-activity-data/

[13] Oliveira RB, Silva EN, Charpinel DMF, et al. Secondary hyperparathyroidism status in Brazil: Brazilian Census of Parathyroidectomy. J Bras Nefrol. 2011;33:457–462. 22189810

[14] Goldwasser RS, Lobo MSC, Arruda EF, et al. Difficulties in access and estimates of public beds in intensive care units in the state of Rio de Janeiro. Rev Saúde Pública. 2016;50:1–9. 27191155 10.1590/S1518-8787.2016050005997 PMC4902093

[15] van der Plas WY, Engelsman AF, Özyilmaz A, et al. Impact of the introduction of calcimimetics on timing of parathyroidectomy in secondary and tertiary hyperparathyroidism. Ann Surg Oncol. 2017;24:15–22. 27459979 10.1245/s10434-016-5450-6 PMC5179588

[16] Nascimento JCP, Brescia MDG, Custódio MR, et al. Early postoperative parathormone sampling and prognosis after total parathyroidectomy in secondary hyperparathyroidism. J Bras Nefrol. 2017;39:135–140. 28489181 10.5935/0101-2800.20170021

[17] Fülöp T, Koch CA, Farah Musa AR, et al. Targeted surgical parathyroidectomy in end-stage renal disease patients and long-term metabolic control: a single-center experience in the current era. Hemodial Int. 2018;22:394–404. 29446565 10.1111/hdi.12639

[18] Tentori F, Wang M, Bieber BA, et al. Recent changes in therapeutic approaches and association with outcomes among patients with secondary hyperparathyroidism on chronic hemodialysis: the DOPPS study. Clin J Am Soc Nephrol. 2015;10:98–109. 25516917 10.2215/CJN.12941213 PMC4284424

[19] Kestenbaum B, Andress DL, Schwartz SM, et al. Survival following parathyroidectomy among United States dialysis patients. Kidney Int. 2004;66:2010–2016. 15496173 10.1111/j.1523-1755.2004.00972.x

[20] Yuen NK, Ananthakrishnan S, Campbell MJ Hyperparathyroidism of renal disease. Perm J. 2016;20:78–83. 10.7812/TPP/15-127 PMC4991918 27479950

[21] Ivarsson KM, Akaberi S, Isaksson E, et al. The effect of parathyroidectomy on patient survival in secondary hyperparathyroidism. Nephrol Dial Transplant. 2015;30:2027–2033. 26374600 10.1093/ndt/gfv334 PMC4832998

[22] Valente-Da-Silva HG, Maya MCA, Moreira AS Parathyroidectomy in chronic kidney disease: effects on weight gain and on quality of life improvement. Rev Col Bras Cir. 2017;44:263–269. 28767802 10.1590/0100-69912017003007

[23] Cheng SP, Lee JJ, Liu TP, et al. Parathyroidectomy improves symptomatology and quality of life in patients with secondary hyperparathyroidism. Surgery. 2014;155:320–328. 24035616 10.1016/j.surg.2013.08.013

[24] Apetrii M, Goldsmith D, Nistor I, et al. Impact of surgical parathyroidectomy on chronic kidney disease-mineral and bone disorder (CKD-MBD) - a systematic review and meta-analysis. PLoS One. 2017;12:e0187025. 29107998 10.1371/journal.pone.0187025 PMC5673225

[25] Chen L, Wang K, Yu S, et al. Long-term mortality after parathyroidectomy among chronic kidney disease patients with secondary hyperparathyroidism: a systematic review and meta-analysis. Ren Fail. 2016;38:1050–1058. 27198474 10.1080/0886022X.2016.1184924

[26] Ng'andu NH An empirical comparison of statistical tests for assessing the proportional hazards assumption of Cox's model. Statist Med. 1997;16:611–626. 10.1002/(sici)1097-0258(19970330)16:6<611::aid-sim437>3.0.co;2-t 9131751

[27] Ma TL, Hung PH, Jong IC, et al. Parathyroidectomy is associated with reduced mortality in hemodialysis patients with secondary hyperparathyroidism. Biomed Res Int. 2015;2015:1. 10.1155/2015/639587 PMC4433652 26064934

